# TRPV2 and TRPC5 are potential targets for astringent phytochemicals

**DOI:** 10.1016/j.crfs.2026.101306

**Published:** 2026-01-10

**Authors:** Anna Kadkova, Kamila Kosinova, Marketa Klouckova, Dita Strachotova, Ivan Barvik, Lucie Zimova, Viktorie Vlachova

**Affiliations:** a Institute of Physiology, Czech Academy of Sciences, Prague, Czech Republic; b Faculty of Mathematics and Physics, Institute of Physics, Charles University, Prague, Czech Republic

**Keywords:** Transient receptor potential, TRP channel, Astringency, Genistein, Theasinensin A, Tannic acid

## Abstract

Astringency is a multimodal sensory experience resulting from complex interactions between chemical compounds and the oral environment, involving tactile, chemosensory and thermosensory pathways. Recent human studies have examined the role of the polymodal transient receptor potential (TRP) channels TRPV1 and TRPA1 in astringency perception; however, other thermo- and mechanosensitive TRP channels expressed in oral epithelial cells and in trigeminal neurons innervating the mouth and tongue may also contribute to this complex sensation. This study explored the effects of structurally distinct representatives of astringent compounds on TRPV2 and TRPC5 channels. Using patch-clamp electrophysiology, microfluorimetry, molecular modeling, and mutagenesis, we show that the auto-oxidation products of the most abundant green tea polyphenol (−)-epigallocatechin-3-gallate (oxi-EGCG) significantly increase the activation of rat TRPV2 while blocking the human orthologue. The plant-derived isoflavone genistein, but not its glycoside form genistin, potentiated human TRPV2 and sensitized TRPC5-mediated currents activated by depolarizing voltage and the alpha subunit of G-proteins. Tannic acid, another astringent substance, potentiated rat TRPV2 and inhibited human TRPV2 and TRPC5. Furthermore, we show that both channels can interact with mucin 1, a transmembrane glycoprotein present in the native oral environment. Our data also provide the first evidence of heat-induced activation of human TRPV2. Considering previous evidence for TRPV2 and TRPC5 expression in the oral cavity and their roles in oral pain and cancer, our findings indicate that these polymodal channels may participate not only in detecting specific astringent compounds, but also in mediating their broader health-related and anesthetic actions.

## Highlights

• TRPV2 and TRPC5 ion channels are modulated by different types of astringents.
• Astringents exhibit different effects on rat and human TRPV2 orthologues.
• Genistein, but not its glycoside form genistin, potentiates TRPV2 and TRPC5.
• Both channels can interact with the transmembrane glycoprotein mucin 1.

## Graphical abstract

**Figure:**
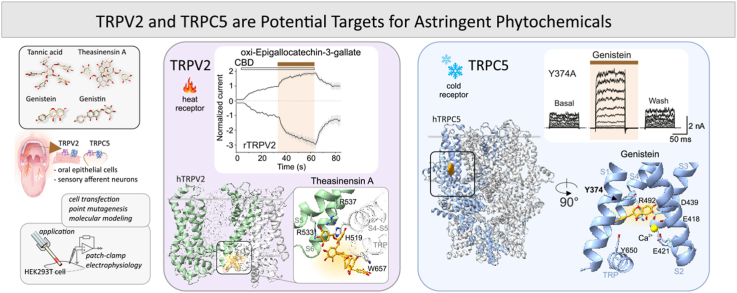

## 1 Introduction

Astringency, a complex multimodal sensation, involves various sub-qualities mediated by multiple mechanisms at the molecular level. Although traditionally considered a tactile sensation associated with a loss of salivary lubricity, salivary protein aggregation and increased oral friction (Bajec and Pickering, 2008; Breslin et al., 1993; Canon et al., 2021), current research suggests that astringency also involves chemosensory mechanisms including several types of receptors and sensory cells in the oral cavity that coordinate mechanical, chemical, and possibly inflammatory processes (Huang and Xu, 2021; Pires et al., 2020; Wang et al., 2024; Wei et al., 2023). The exact chemosensory bases of astringency detection and the nature of the activated receptors remain largely unknown. Similar to pungency, astringency can be seen as an external signal that warns the organism of the potential danger of certain foods, triggering a response that prevents, or limits, their consumption. It is exactly for these situations that living organisms are equipped with receptors in the peripheral tissues that recognize even weak stimuli in a timely manner, convert them into depolarization of sensory neurons, and reliably inform the central nervous system (Bamps et al., 2021). Such a role is played in particular by polymodal ion channels from the transient receptor potential (TRP) family expressed in somatosensory nerve endings, which are inactive under normal conditions and are activated when a physiological threshold is exceeded (Bamps et al., 2021; Clapham, 2003; Julius, 2013; Rosenbaum et al., 2022). In addition to chemosensation, these channels detect and transduce stimuli across various modalities, such as noxious heat, cold, osmotic stress, mechanical stretch, membrane depolarization or UV light (Koivisto et al., 2022; Nilius and Szallasi, 2014). Typically, the combined effects of the multiple TRP stimuli are not additive, and their synergy greatly increases the response effectiveness (Baez-Nieto et al., 2011; Fernández-Ballester et al., 2023).

The earliest studies proposing a role for polymodal TRP channels in astringency perception investigated whether polyphenolic compounds, known to cause this sensation, could activate these channels *in vitro*. Using human immortalized oral epithelial cells (TR146), the authors found that while these cells responded to the respective agonists of TRPV1, TRPV3 and TRPM8, capsaicin, camphor and menthol, they did not respond to black tea solutions rich in polyphenols (Carpenter, 2013). On the other hand, TRPV1 and TRPA1 were activated in sensory neurons and intestinal enteroendocrine cells by auto-oxidation products of epigallocatechin gallate, the most abundant green tea polyphenol that produces a strong astringency (Kurogi et al., 2012, 2015). The diversity found in the sensitivity of TRPV1 and TRPA1 orthologues to polyphenolic astringents indeed suggested an evolutionary relationship between TRP proteins expressed in different animal species and their feeding behaviors (Takahashi et al., 2021). In 2024, Kim and Simons investigated the role of TRPV1 and TRPA1 channels in astringency perception by desensitizing these receptors in human subjects using capsaicin and mustard oil, respectively (Kim and Simons, 2024). Participants then assessed the astringency of four distinct compounds: epicatechin, epigallocatechin gallate (EGCG), tannic acid, and potassium alum. The study found no significant differences in astringency perception between desensitized and control conditions, suggesting that TRPV1 and TRPA1 may not play a pivotal role. However, since astringency is mainly related to oral tactile sensitivity, greater focus on mechanically sensitive members of the TRP channel family, many of which have not yet been studied in this context, could help clarify the mechanisms involved. Among these channels, TRPV2 is expressed in both oral epithelial cells and trigeminal neurons innervating the mouth and tongue (Chalazias et al., 2022; Oto et al., 2022; Sakakibara et al., 2017; Sasaki et al., 2013; Shimohira et al., 2009; Siveen et al., 2020; Urata et al., 2018; Wang et al., 2023). Functional TRPV2 channels are likely active in trigeminal neurons, contributing to mechanical and heat sensation (Shimohira et al., 2009; Urata et al., 2018). Expression has also been reported in oral epithelial and dendritic cells (Sasaki et al., 2013; Shimohira et al., 2009; Wang et al., 2011), although its functional significance remains less well defined. Recent research suggests that TRPV2 could serve as a biomarker for oral squamous cell carcinoma and may be involved in mechanisms driving oral tumor development and progression (Sakakibara et al., 2017; Siveen et al., 2020).

Another mechanosensitive TRP channel, TRPC5, is expressed in small- and medium-sized trigeminal neurons, including those that innervate the oral cavity and tongue (Bernal et al., 2021; Zimmermann et al., 2011). A recent study by Bernal et al. (2021) provides evidence for the expression of TRPC5 in the trigeminal ganglia, specifically in the mandibular and maxillary branches, and highlights its significance in oral sensory transduction (Bernal et al., 2021). Several natural flavonols with moderate-to-strong astringent properties, such as kaempferol, galangin and quercetin, inhibit TRPC5 activity, whereas the flavone apigenin (lacking a hydroxyl group at the 3-position of the central ring) activates the channel (Naylor et al., 2016). All these compounds are extensively studied for various biological activities, including antioxidant, anti-inflammatory, antibacterial, and anticancer effects. The implication of TRPC5 in the progression and chemoresistance of various cancers (He and Ma, 2016; Yu et al., 2024) suggests, as in the case of TRPV2, that studying the effects of astringents on these channels may have important implications for oral oncology.

In this study, we examined the effects of exemplary, structurally distinct representatives of astringent compounds on heterologously expressed TRPV2 and TRPC5 to evaluate their potential role in the perception of astringency. The polymodal nature of the activation of these channels may prompt a novel direction of research into mechanical, chemo- and thermosensory pathways of astringent transduction, providing a more refined understanding of how we perceive this complex sensation.

## 2 Materials and methods

### 2.1 Cell culture and transfection

Human embryonic kidney 293T cells (HEK293T, ATCC) were cultured in Opti-MEM I medium (Thermo Fisher Scientific) supplemented with 5 % fetal bovine serum (PAN-Biotech). Before transfection, cells were placed into 24-well plates coated with poly-l-lysine and collagen. After reaching ∼70 % confluency, cells were transiently co-transfected with 200 ng of eGFP plasmid (in the pcDNA3.1 vector) and with 300 ng of plasmid encoding wild-type or mutant human TRPC5 or TRPV2 (rat or human; pCMV6-XL5; OriGene Technologies, Rockville, MD, USA) using the magnet-assisted transfection technique (PolyMag Neo, OZ Biosciences). In specific cases, an expression plasmid encoding human MUC1 (pReceiver-M56; GeneCopoeiaTM; Catalog No.: EX-Z3132-M56) was added to the transfection mixture at a 1:2 or 3:2 (TRPC5:MUC1 or TRPV2:MUC1) ratio; 600 ng or 200 ng). Cells were then plated on poly-l-lysine-coated glass coverslips. Electrophysiological recordings were performed 24–48 h after transfection. At least two independent transfections were used for each experimental group. The wild-type TRPC5 and TRPV2 channels were regularly tested alongside the experiments.

### 2.2 Patch-clamp electrophysiology

Whole-cell membrane currents were recorded with an Axopatch 200B amplifier and the software pCLAMP 10 (Molecular Devices, Sunnyvale, CA, USA). Data were filtered at 2 kHz using the low-pass built-in 8-pole Bessel filter and digitised at 5–10 kHz using the Digidata 1550B analog-to-digital converter controlled by Clampex 10 (Molecular Devices). Patch pipettes were prepared from borosilicate glass capillaries with 1.5-mm outer diameter (Science Products, Hofheim, Germany) pulled on a horizontal puller P-87 (Sutter Instrument, Novato, CA, USA) and heat-polished with microforge MF-83 (Narishige, Tokyo, Japan) to a final resistance 3–5 MΩ. Two voltage stimulation protocols were used; 100 ms voltage steps from −80 to +200 mV (with +20 mV increments) applied from a holding potential of −70 mV, and voltage ramps from −100 mV to +100 mV in 500 ms (0.4 V s^−1^) applied every 1 s from a holding potential of 0 mV. The liquid-junction potential was calculated to be +4.9 mV using Clampex 10 software; data were not corrected for this offset. The recordings were performed at room temperature (22–25 °C). Only one recording was performed on any one coverslip of cells to ensure that recordings were made from cells that had not been previously exposed to chemical stimuli.

### 2.3 Heat stimulation

A system for rapid superfusion of the cultured cells was used for thermal stimulation and drug application (Dittert et al., 2006). Briefly, experimental solutions were driven by gravity from seven to ten different barrels, through automatically controlled valves, to a manifold that consisted of fused silica tubes connected to a common outlet glass capillary. The lower part of the outlet capillary was wrapped with densely coiled copper wire that heated the solution to a chosen temperature. Voltage commands for heat and drug stimulation were generated from the Digidata 1440 digitizer using pCLAMP 10 software (Molecular Devices, Sunnyvale, CA, USA). Ramp-shaped temperature increases from room temperature to >50 °C within 10 s were applied at 30 s intervals. The volume of solution in the experimental dish and the immersion of the application capillary were maintained at a constant level, and an internal table generated after balancing an electrical circuit of the system was utilized, which resulted in a good reproducibility of the heat stimuli.

### 2.4 Experimental solutions

The extracellular solution (ECS) before the whole-cell recording contained: 160 mM NaCl, 2.5 mM KCl, 1 mM CaCl_2_, 2 mM MgCl_2_, 10 mM HEPES, 10 mM glucose; pH adjusted to 7.3 with NaOH, 310 mosmol·l^−1^. The extracellular control bath solution for recording contained: 150 mM NaCl, 5 mM EGTA, 10 mM HEPES, 300 mosmol·l^−1^, adjusted to pH 7.4 with NaOH. The Cs-rich solution contained: 140 mM CsCl, 1 mM CaCl_2_, 2 mM MgCl_2_, 10 mM HEPES and 10 mM glucose; adjusted to pH 7.3 with CsOH, 280 mosmol·l^−1^. The glass pipettes were filled with intracellular solution containing: 145 mM CsCl, 3 mM CaCl_2_, 2 mM MgATP, 10 mM HEPES, 5 mM EGTA; pH adjusted to 7.3 with CsOH, 300 mosmol·l^−1^. EGCG was dissolved in ECS at 100 μM and incubated at 25 °C for 1–6 h or freshly prepared just before use. Tannic acid was dissolved in ECS at 10 μM and freshly prepared before use. Cannabidiol (CBD), genistein and genistin were dissolved in DMSO and stored as 10 mM (for CBD) or 100 mM aliquots at −20 °C. Before adding to cells, the compounds were diluted in the extracellular bath solution to a concentration of 30 μM (CBD) or 100 μM (genistein, genistin), resulting in a final DMSO concentration of ∼0.3 %/0.1 %. Reagents were purchased from Merck Life Science unless stated otherwise.

### 2.5 Ca^2+^ imaging

Imaging was performed 24–48 h after cell transfection. Cells were loaded with Fura 2-AM (10 μM, Calbiochem, CA) for 45 min and subsequently washed and imaged in standard bath solution containing (in mM): 140 NaCl, 5 KCl, 2 CaCl_2_, 2 MgCl_2_, 10 HEPES, pH 7.4. Ratiometric Ca^2+^-imaging was performed on the Cell^R imaging system consisting of an Olympus IX81 microscope, Polychrome V polychromator (Till Photonics, Planegg, Germany), Hamamatsu Orca-ER camera (Hamamatsu Photonics, Hamamatsu City, Japan), and the Cell^R imaging software (Olympus Biosystems, Planegg, Germany). Fura-2 emission images (at >510 nm) were obtained with exposures of 300 ms at 340 nm and 100 ms at 380 nm excitation wavelengths. The ratio of the fluorescence intensity obtained at 340 nm and 380 nm was used to determine the Ca^2+^ signal at 1 s intervals.

### 2.6 Live-cell imaging and acceptor bleaching

The cells were transfected and grown in a 3.5 cm culture dish with glass bottom (Cellvis, Mountain View, CA, USA). Cells were observed in the inverted IX83 microscope equipped with a FV1200 confocal scanner (Olympus, Germany) using 561 nm excitation for mCherry fluorescence and 488 nm excitation for eYFP and for differential interference contrast (DIC). UPLSAPO 60XW NA = 1.2 water-immersion objective (Olympus) was used for imaging. During monitoring at room temperature (21 °C), the culture dish were placed in cell cultivation chamber with continuous supply of 5 % CO_2_ (OKOLAB). Fluorescence images were processed by the FluoView software FV10-ASW 3.1 and ImageJ-FiJi software (FiJi software, Bethesda, USA). Acceptor photobleaching was done by a 561 nm semiconductor CW laser.

### 2.7 FLIM - fluorescence lifetime imaging and lifetime data processing

The apparatus used for lifetime imaging as well as lifetime data processing, is described in detail elsewhere (Herman et al., 2019; Holoubek et al., 2021). Specifically, in this study, fluorescence was excited by a pulsed diode laser (LDH-DC-485, 485 nm, PicoQuant) running at a 20 MHz repetition rate. Light was coupled to the inverted IX83 microscope by a single-mode optical fiber and reflected to the sample by a 488 nm long-pass dichroic mirror (Olympus). UPLSAPO 60XW NA 1.2 water-immersion objective (Olympus) was used for imaging. Fluorescence was directed via multimode optical fiber to a cooled GaAsP hybrid PMT (PicoQuant) through the 534/30 bandpass filter (Semrock). Signal was processed by the TimeHarp 260-PICO TCSPC card and the SymPhoTime64 software (both PicoQuant). To avoid pile-up artifacts, the data collection rate at brightest pixels was kept below 5 % of the excitation frequency. Cells with membrane localization of MUC1 and TRPC5 or TRPV2 were evaluated.

### 2.8 Molecular docking

Molecular predictions of different interaction sites for theasinensin A (TSA), tannic acid and genistein at human TRPC5, rat and human TRPV2 were performed using Boltz-2, the first deep learning model approaching the accuracy of physics-based free-energy perturbation (FEP) methods (Passaro et al., 2025). Docking of TSA to rat TRPV2 was performed using AutoDock Vina (Trott and Olson, 2010). The initial coordinates of TSA were taken from PubChem (Identifier CID 442543) and minimized using Tools -Structure Editing - Minimize Structure tool in UCSF Chimera 1.18 (Pettersen et al., 2004). Docking was carried out over a search space of 100 × 100 × 100 Å that covers almost the entire channel except for the terminal cytoplasmic parts. 78 out of the 80 output poses exhibited conformations located within the pocket formed by the positively charged residues H521, R535, and R539, located at the intracellular interface between S5 and the S4–S5 linker of two monomers. The two remaining output positions were located inside the channel pore. The docking results from AutoDock Vina are shown in Supplementary Fig. 4A and B. The results from molecular modeling were visualized and analysed with UCSF Chimera 1.18, ChimeraX 1.10 and Yasara 22.9.24 software.

### 2.9 Analysis of data

**Fig. 1:**
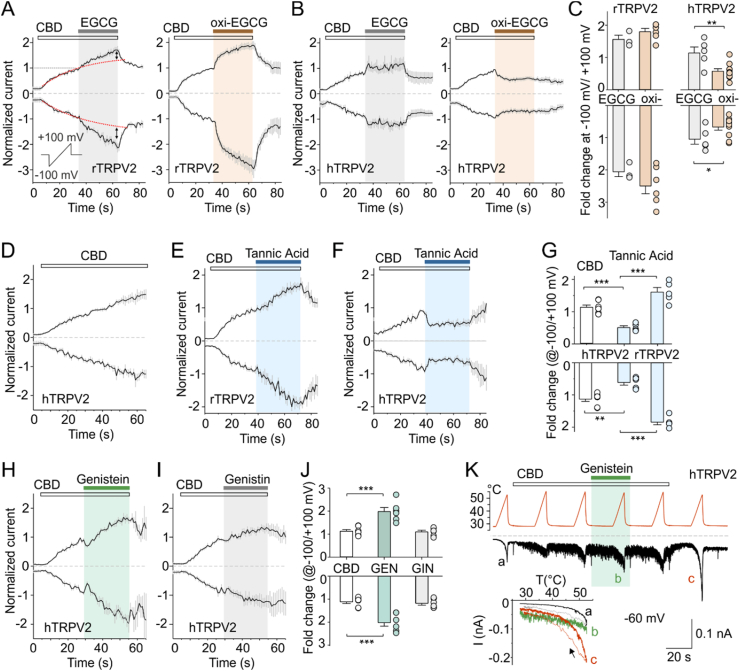
| Astringent compounds modulate activity of rat and human TRPV2**. A** Average whole-cell currents recorded from HEK293T cells expressing rat TRPV2 (rTRPV2), measured at −100 mV and +100 mV. Cannabidiol (CBD; 30 μM) was pre-applied prior to its co-application with epigallocatechin gallate (EGCG; 100 μM; *n* = 3) or its auto oxidation products (oxi-EGCG; 100 μM; incubated for longer than 70 min; *n* = 6). A ramp pulse was periodically applied every 1 s for 500 ms (shown in the inset). Currents were normalized to maximum amplitudes elicited after 30s application of CBD (horizontal dotted line). **B** Average whole-cell currents recorded from HEK293T cells expressing human TRPV2 (hTRPV2), measured at −100 mV and +100 mV. Cannabidiol (CBD; 30 μM) was pre-applied prior to its co-application with epigallocatechin gallate (EGCG; 100 μM; *n* = 5) or its auto oxidation products (oxi-EGCG; 100 μM; *n* = 10). **C** Relative change in peak currents after 30s application of EGCG or oxi-EGCG versus current extrapolated by fitting the CBD currents with a single-exponential function (indicated by red dashed line and vertical double arrows in the panel A). The current amplitudes were determined 30 s after application of the CBD mixture with EGCG or oxi-EGCG and compared with the amplitude values extrapolated using an exponential function determined within the application interval of CBD alone. **D** Average whole-cell currents recorded from HEK293T cells expressing human TRPV2, measured at −100 mV and +100 mV. Cannabidiol (CBD; 30 μM) was applied for 60 s (*n* = 7). **E and F** Average whole-cell currents recorded from HEK293T cells expressing rTRPV2 (E; *n* = 5) and hTRPV2 (F; *n* = 5), measured at −100 mV and +100 mV. Cannabidiol (CBD; 30 μM) was pre-applied prior to its co-application with tannic acid (100 μM); **G** Relative increase in peak currents after 30s application of tannic acid versus current extrapolated by fitting the CBD currents with a single-exponential function. **H and I** Average whole-cell currents recorded from a HEK293T cell expressing human TRPV2, measured at −100 mV and +100 mV. Cannabidiol (CBD; 30 μM) was pre-applied prior to its co-application with genistein (100 μM; H; *n* = 6) or its glycosylated derivative genistin (100 μM; I; *n* = 6). **J** Relative change in peak currents after 30s application of genistein (GEN) and genistin (GIN) versus current extrapolated by fitting the CBD currents with a single-exponential function. **K** Representative whole-cell current of human TRPV2 expressed in HEK293T cells elicited by 10s heat ramps (from 27 °C to 55 °C) repeated in 30s intervals in extracellular bath solution at holding potential of −60 mV. Cannabidiol (CBD; 30 μM) was applied for 60 s followed by its co-application with genistein (100 μM) for 30 s. Neither the presence of CBD nor the mixture of CBD and genistein sensitized the human TRPV2 to heat (data point b). After CBD washout, the heat-induced current increased (data point c). Inset: Current-temperature relationship in indicated times (a, b and c). ∗*P* < 0.05, ∗∗*P* < 0.01 and ∗∗∗*P* < 0.001, Student's two-tailed *t-*test. Error bars represent standard error of the mean (SEM).

Electrophysiology data were analysed using Clampfit 10 and 11 (Molecular Devices, San Jose, CA, USA), SigmaPlot 10 (Systat Software Inc., San Jose, CA, USA) and OriginPro 2021 (OriginLab Corporation, Northampton, MA, USA). Voltage-dependent gating parameters were estimated from steady-state conductance-voltage (*G*/*V*) relationships obtained at the end of 100-ms voltage steps by fitting the conductance *G* = I/(*V*−*V*_rev_) as a function of the test potential *V* to the Boltzmann equation: *G* = ((*G*_max_ − *G*_min_)/(1 + exp[−*zF*(*V*−*V*_50_)/*RT*])) + *G*_min_, where *z* is the apparent number of gating charges involved in channel opening (in elementary charge units: *e*_o_ = 1.6 × 10^−19^ C), *V*_50_ is the half-activation voltage, *G*_min_ and *G*_max_ are the minimum and maximum whole-cell conductance, *V*_rev_ is the reversal potential, and *F*, *R*, and *T* have their usual thermodynamic meaning. To evaluate changes in TRPV2-mediated currents, we fitted a one-exponential function (*y* = *A*e^*-t/τ*^ + *C*) to the initial 30 s of the amplitudes measured at −100 mV and +100 mV in the presence of CDB alone and extrapolated the fits to the following 30 s (as indicated by the red dotted line in Fig. 1A). Amplitudes measured in the presence of tested compounds were related to the extrapolated values. Throughout, average data are presented as means ± standard error of the mean (SEM), or as a median, range, and interquartile range as appropriate. The heat-evoked whole cell currents sampled during the rising phase of temperature ramp were pooled for every 0.25 °C. The maximum temperature coefficients *Q*_10_ and the thermal thresholds were determined from the slope of Arrhenius plot (absolute values of inward currents plotted on a logarithmic scale, y-axis, against the reciprocal of the absolute temperature, x-axis) as described previously (Macikova et al., 2019; Vlachova et al., 2003). The lower and upper limits for *Q*_10_ estimation were defined as the temperatures at which the fit of the Arrhenius plot declined significantly from a straight line (*r*^2^ < 0.98). Statistical significance was calculated using Student's *t*-test, Mann-Whitney rank-sum, or one-way analysis of variance followed by the non-parametric Dunn's test, as appropriate. Differences were considered significant at *P* < 0.05.

## 3 Results

### 3.1 Auto-oxidized products of epigallocatechin gallate stimulate rat TRPV2

Epigallocatechin gallate (EGCG) is one of the most astringent catechins present in green tea (Li et al., 2018). When EGCG undergoes auto-oxidation (by incubating in a buffered salt solution for 3 h), it forms compounds that significantly activate both TRPA1 and TRPV1 in human embryonic kidney (HEK) 293 cells (Kurogi et al., 2015), while freshly prepared EGCG is inactive. To investigate whether EGCG or auto-oxidized EGCG (oxi-EGCG) can affect other polymodal TRP channels, we measured membrane currents from HEK293T cells overexpressing rat (rTRPV2) or human TRPV2 (hTRPV2) by whole-cell electrophysiology. Voltage ramps of 500 ms duration from −100 mV to +100 mV were delivered periodically every 1 s from a holding potential of 0 mV to record the outward and inward currents developed over time (Fig. 1A–D). The experiments were performed in Ca^2+^-free conditions to exclude the influence of Ca^2+^ on TRPV2 (Mercado et al., 2010). EGCG, freshly prepared at a concentration of 100 μM, did not produce any significant currents in cells expressing rTRPV2 or hTRPV2 (Supplementary Fig. 1A) but affected currents induced by the non-selective agonist cannabidiol (CBD; 30 μM) (Fig. 1A). CBD activation of hTRPV2 was slow and did not reach saturation even after 1 min (Fig. 1D). Therefore, we chose a fixed interval of 30 s for CBD application and the effects of the tested compounds co-applied together with CBD for the following 30 s were compared with the extrapolated amplitude that would theoretically be achieved in the presence of CBD alone (see Materials and Methods, Fig. 1A–D and Supplementary Fig. 1C–F). A statistically significant increase after 30 s of the CBD and ECGC co-application was detected in rTRPV2 at both positive and negative membrane potentials (*P* = 0.022 and 0.029; paired *t*-test; *n* = 3). When EGCG was incubated in a buffered extracellular control solution for longer than 70 min, its co-application with CBD resulted in a marked increase in currents measured from cells expressing rTRPV2 at both membrane potentials (*P* = 0.002; paired *t*-test; *n* = 6) (Fig. 1A–C, Supplementary Fig. 1B). Freshly prepared EGCG had no significant effect on CBD-induced currents mediated by hTRPV2 (*P* = 0.813; paired *t*-test; *n* = 5), whereas auto-oxidized EGCG efficiently inhibited the channels at both positive and negative potentials (*P* = 0.012 and 0.038; paired *t*-test; *n* = 10) (Fig. 1B and C). In Ca^2+^ imaging experiments, the rTRPV2-transfected cells displayed a small increase in intracellular calcium concentration ([Ca^2+^]_i_) during oxi-EGCG applications (100 μM; 2-min duration), but changes were not detectable in hTRPV2-expressing cells (Supplementary Fig. 2A and B). As a control, we confirmed the previously demonstrated sensitivity to oxi-EGCG in rat TRPV1 (Kurogi et al., 2015). In addition, we also detected a significant increase in [Ca^2+^]_i_ in response to oxi-EGCG in cells expressing human TRPC5 (Supplementary Fig. 2C and D), but subsequent whole-cell recordings showed no significant effect (Supplementary Fig. 1I).

### 3.2 Tannic acid potentiates rat TRPV2 but blocks its human orthologue

Tannic acid is a polyphenolic compound that is present naturally in many plants, especially in leaves, tree bark and unripe fruits. At a concentration of 30 μM, this astringent compound stimulates the influx of Ca^2+^ in brain endothelial cells, in which the involvement of TRPM4 and TRPC4/C5 channels has recently been suggested (Tsai et al., 2020). We explored the effect of tannic acid on rat and human TRPV2 expressed in HEK293T cells using a voltage ramp protocol, as in the previous experiment. As shown in Fig. 1E–G, tannic acid at a concentration of 10 μM potentiated CBD-induced activity in rat TRPV2 but inhibited the activity of the human orthologue. Tannic acid alone had no significant effect on cells expressing rTRPV2 or hTRPV2 (Supplementary Fig. 1G). These results indicate that, similar to oxi-EGCG, there is a species-specific difference between the rTRPV2 and hTRPV2 orthologues. This suggests that the channel's sensitivity to these astringent compounds has not been evolutionarily conserved or its physiological significance may be limited. Although hTRPV2 does not appear to play a role as a primary excitatory channel informing the sensory system about the astringency of oxi-EGCG or tannic acid, it is evident that these substances can modulate the function of the channel, with the characteristics of their effects depending on the channel's activation state.

### 3.3 Genistein potentiates rat and human TRPV2

Genistein is a prominent isoflavone found in soybeans that contributes to both the bitterness and astringency experienced in soy-based products (Karolkowski et al., 2023). At relatively low concentrations, genistein activates human bitter taste receptors hTAS2R14 and hTAS2R39 (the half maximal effective concentration, EC_50_, values of 29 and 49 μM), leading to subsequent activation of signaling pathways involving phospholipase C β2 and the release of intracellular Ca^2+^ (Nowak et al., 2018; Roland et al., 2011). At higher concentrations, genistein may also impart a pronounced astringent sensation under some conditions, including the presence of metal ions, which can complex with it (Dowling et al., 2010; Singha Roy et al., 2013), altering its solubility and enhancing astringency. We found that genistein (100 μM) significantly enhanced responses of rat TRPV2 induced by 300 μM 2-APB (Supplementary Fig. 1H). However, the human TRPV2 orthologue is essentially insensitive to 300 μM 2-APB (Fricke et al., 2025; Juvin et al., 2007; Neeper et al., 2007), so we used cannabidiol as an agonist. To investigate the effects of genistein on human TRPV2, we applied it at a concentration of 100 μM either alone or in combination with cannabidiol (CBD; 30 μM); as in previous experiments, voltage ramps were delivered from +100 to −100 mV every 1 s from a holding potential of 0 mV. The same protocol was followed to test the effect of genistin (100 μM), the glycosylated form of genistein, which has a glucose molecule attached to the hydroxyl group at position 7 (Supplementary Fig. 5D). Genistein significantly potentiated hTRPV2-mediated currents when CBD was pre-applied for 30 s prior to its co-application with genistein (Fig. 1H). After 30 s of exposure, genistein potentiated CBD-induced responses at positive and negative membrane potentials (2.0 ± 0.2 fold at −100 mV and +100 mV; *n* = 6). In contrast, genistin had no significant effect on CBD-induced responses (Fig. 1I and J). Administration of genistein alone did not affect cells expressing rTRPV2, hTRPV2, or related human TRPV3 channels, as determined by Ca^2+^ imaging experiments and electrophysiology (Supplementary Fig. 2E–G). Interestingly, in hTRPV2-expressing cells, 2-APB (100 μM) increased [Ca^2+^]_i_ after application of cannabidiol and genistein (Supplementary Fig. 2H and I), indicating that hTRPV2 retains sensitivity to 2-APB. We further investigated whether genistein is capable of sensitizing hTRPV2 to heat (Fig. 1K). The human TRPV2 orthologue is generally considered to be essentially insensitive to heat up to 60 °C (Fricke et al., 2019; Fricke and Leffler, 2024; Neeper et al., 2007). However, in our hands, a heat ramp from 27 °C to 55 °C produced a small but apparently specific hTRPV2-mediated response at a holding potential of −60 mV, as the currents showed a steep temperature dependence above ∼52 °C and clear hysteresis upon cooling (see inset in Fig. 1K and Supplementary Fig. 3A and B). The presence of CBD strongly increased the noise of the recording, confirming the specific activity of the channels. The addition of genistein had no apparent effect on CBD- and heat-induced activity. After washing, the heat-induced currents increased significantly, suggesting that hTRPV2 likely shares the use-dependence of heat activation with its rat orthologue (Liu and Qin, 2016). The specific strong temperature dependence of hTRPV2-mediated responses was consistently detected under various experimental conditions in which the temperature exceeded 55–60 °C (Supplementary Fig. 3). These high temperatures clearly led to irreversible changes in the channel protein and loss of the high temperature threshold for activation, as has recently been described for rTRPV1 (Mugo et al., 2023) and rTRPV2 (Mugo et al., 2025). Together, these data indicate that, similar to rTRPV2 (Gochman et al., 2023), human TRPV2 is not sensitized to heat in the presence of CBD. Moreover, our data clearly demonstrate, for the first time, to our knowledge, that high temperatures above ∼55–60 °C activate specific hTRPV2-mediated currents exhibiting high *Q*_10_ values (>20; Supplementary Fig. 3C).

### 3.4 Molecular binding modes of different astringents at TRPV2

**Fig. 2:**
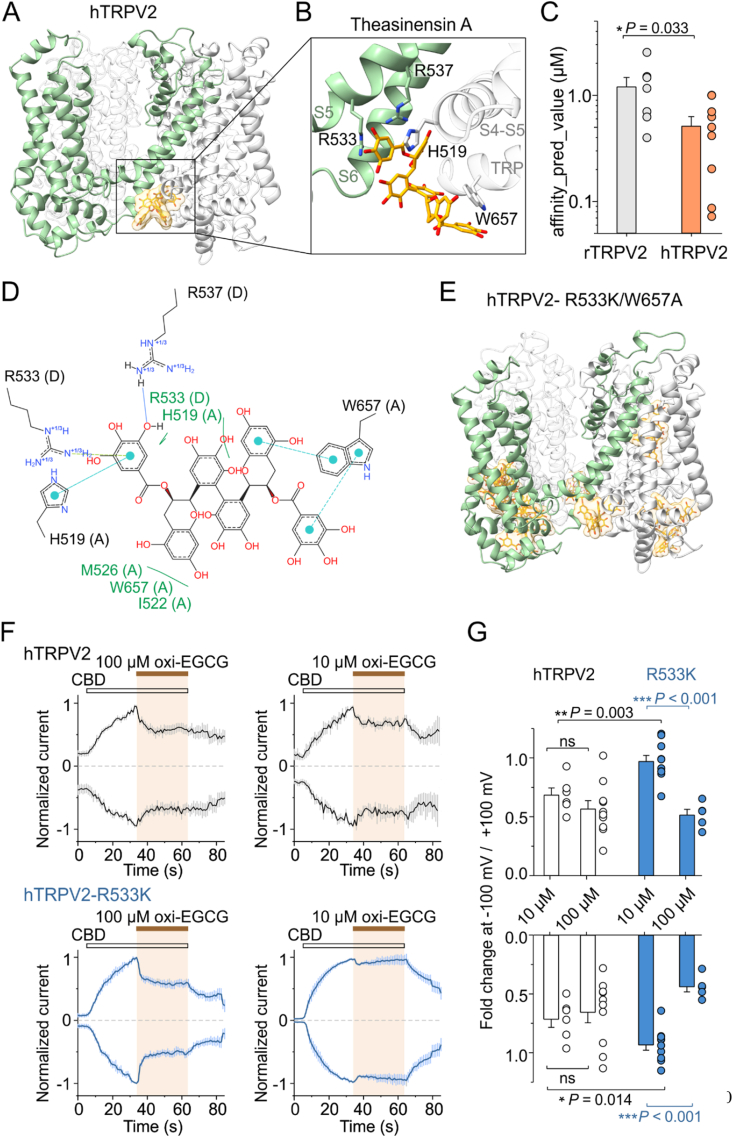
| Predicted binding site for the main auto-oxidation product of EGCG, theasinensin A, in human TRPV**2. A and B** Theasinensin A (TSA) binds beneath the presumed inner leaflet of the plasma membrane, coordinated predominantly by H519 from the N-terminus of the S4-S5 linker, W657 from the TRP helix, and R533 from the S5 helix of the adjacent subunit (predicted by Boltz-2). **C** The average predicted binding affinity (*IC*_50_) for TSA in rat and human TRPV2 orthologues. rTRPV2 shows lower affinity to TSA than hTRPV2. **D** A detail of the TSA binding site in the hTRPV2 channel. Hydrogen bonds depicted in blue dashed line, π-πinteractions in cyan dashed line, cation-π interaction in light green dashed line and hydrophobic contacts in green. Adjacent subunits of hTRPV2 are denoted by (A) and (D). Created by PoseView (Proteins*Plus* Structure-Based Modeling Support Server). **E** The double mutation R533K/W657A displaced TSA from the binding site to other parts of the hTRPV2 channel complex. **F** Average normalized whole-cell currents recorded from a HEK293T cell expressing wild-type hTRPV2 and R533K mutant of hTRPV2, measured at −100 mV and +100 mV. Cannabidiol (CBD; 30 μM) was pre-applied prior to its co-application with oxi-epigallocatechin gallate at two different concentrations (100 μM; *n* = 10 and 5, and 10 μM; *n* = 6 and 10). **G** Relative increase in peak currents after 30s application of 100 μM or 10 μM oxi-EGCG versus current extrapolated by fitting the CBD currents with a single-exponential function. ∗*P* < 0.05, ∗∗*P* < 0.01 and ∗∗∗*P* < 0.001, Student's two-tailed *t-*test. Error bars represent standard error of the mean (SEM).

To investigate the binding capacities of the explored astringent compounds to TRPV2, we used the recently developed deep learning model Boltz-2, which is capable of not only predicting protein-ligand interactions but also evaluating free energy changes, allowing for the comparison of binding affinities (Wohlwend et al., 2025; Passaro et al., 2025). In specific cases, the results obtained by Boltz-2 were compared to those of AutoDock Vina (Trott and Olson, 2010). This unbiased approach allowed us to predict different interaction sites for theasinensin A (TSA), tannic acid (TA) and genistein at rat and human TRPV2 (Fig. 2 and Supplementary Fig. 4).

First, we explored the binding modes of TSA dimer, which was previously identified as the main oxidation product of EGCG activating TRPA1 and TRPV1 channels (Kurogi et al., 2015). Using Boltz-2, we found that TSA binds invariably to hTRPV2 immediately beneath the presumed inner leaflet of the plasma membrane, coordinated predominantly by H519 from the N-terminus of the S4-S5 linker, W657 from the TRP helix, and R533 from the S5 helix of the adjacent subunit (Fig. 2A–D). The average predicted binding affinity (*IC*_50_) for TSA estimated by Boltz-2 from nine independent runs was 0.5 ± 0.1 μM. A homologous interaction site with the predominant contacts involving H521, R535, and W657 was also consistently predicted for the rat orthologue, which showed significantly lower affinity for TSA than hTRPV2 (1.2 ± 0.3 μM; *P* = 0.033; *n* = 7). In 5 out of 10 independent predictions, the double mutation R533K/W657A displaced TSA from the binding site heterogeneously to other parts of the hTRPV2 channel complex (Fig. 2E) and significantly lowered the predicted probability that TSA is a binder (from 0.60 ± 0.02 to 0.52 ± 0.03; *P* = 0.031, *n* = 10). The single R533K mutation led to the displacement of TSA from the vicinity of the original binding site in 4 out of 10 independent predictions. A corresponding interaction site was also predicted by Autodock Vina using the CBD-bound full-length structure of rat TRPV2, state 1 (PDB ID: 6U8A)(Supplementary Fig. 4A and B). To verify the relevance of the predictions, we expressed either wild-type hTRPV2 or the R533K-hTRPV2 mutant in HEK293T cells and measured the effects of two different concentrations of oxi-EGCG on CBD responses using electrophysiology (Fig. 2F and G). While no difference in the degree of inhibition mediated by 10 μM and 100 μM oxi-EGCG was observed in wild-type hTRPV2 at both negative and positive membrane potentials, the R533K mutant showed a significantly lower degree of inhibition at 10 μM at both potentials (Fig. 2G). This result supports the involvement of R533 in the inhibition of CBD-induced responses of hTRPV2 by oxi-EGCG.

All molecular binding modes of TA on hTRPV2 predicted by the Boltz-2 method (10 runs) overlapped with the positively charged binding pocket identified for TSA, comprised of H519, R533, R537, and W657 (Supplementary Fig. 4C and D). The average predicted binding affinity for TA estimated from ten independent runs was 0.26 ± 0.03 μM. A homologous interaction site (H521, R535, R539, W657) was also consistently predicted for the rat orthologue (Supplementary Fig. 4D), which showed significantly lower affinity for TA than hTRPV2 (0.35 ± 0.02 μM; *P* = 0.043; *n* = 10). The mean predicted probability that TA is a binder at hTRPV2 and rTRPV2 was 0.64 ± 0.03 and 0.66 ± 0.03 (*P* = 0.241). The pocket formed by the positively charged residues H521, R535, and R539, located at the intracellular interface between S5 and the S4–S5 linker of two monomers of rat TRPV2, is known as a key regulatory site targeted by weak acids and the non-selective agonist 2-APB (Fricke et al., 2025; Haug et al., 2024; Pumroy et al., 2022). Our data indicate that, at physiological pH, the total charge of this pocket can be decisive for TA and TSA sensitivity as well. The data also indicate that TA and TSA may interact with both TRPV2 orthologues at homologous binding sites, but they elicit opposite effects, most likely due to the involvement of other non-homologous regions of the channels. One such region that can be considered is located at the bottom of S6, close to the central pore (amino acid residues 651–655; SVATD vs. HVADN in hTRPV2 and rTRPV2, respectively), which contributes to the binding site and changes its conformation upon channel activation (Pumroy et al., 2022).

Genistein preferentially bound to both rTRPV2 and hTRPV2 within the highly conserved vanilloid binding pocket, interacting with the intracellular end of the S3 helix (Y471/Y469 and L475/L473), S2-S3 linker (F467/F465), S4 helix (L510/L508 and L513/L511), S4-S5 linker helix (I529/I527, I533/I531, L534/L532), S5 helix (L537/L535, L538/L536, L541/L539) and S6 of an adjacent subunit (A628/A628 and L632/L631). The average affinity of genistein estimated by Boltz-2 from five independent runs was 27 ± 2 μM and 26 ± 6 μM for rTRPV2 and hTRPV2 (*P* = 0.893; *n* = 5), indicating rather a weak binding or a decoy (Supplementary Fig. 4E and F). In order to bring the predictions closer to the real situation, the input file for the Boltz-2 contained four molecules of cannabidiol in addition to four molecules of genistein. Cannabidiol bound in rat TRPV2 to its usual binding site (Gochman et al., 2023; Pumroy et al., 2022), physically interacting with genistein in the vanilloid cavity (Supplementary Fig. 4G and H). Genistein may therefore stabilize cannabidiol and promote its stimulation of the ion channel.

### 3.5 Tannic acid inhibits human TRPC5

**Fig. 3:**
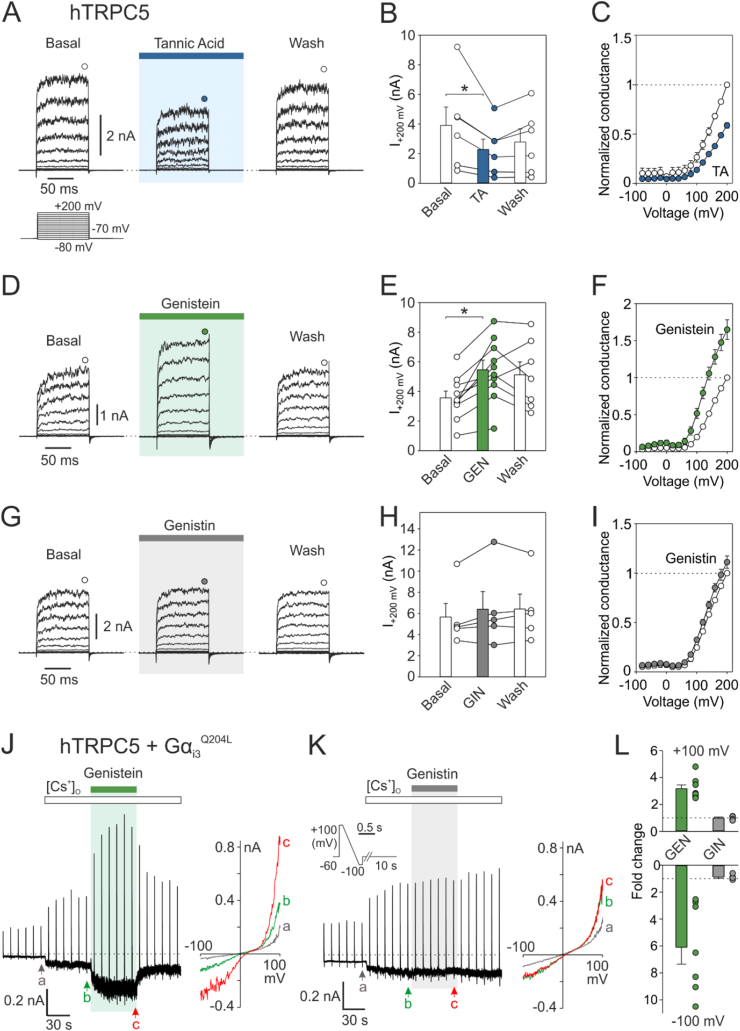
| Astringent compounds modulate activity of human TRPC5**. A** Representative current traces in response to a 100-ms voltage step protocol from −80 to +200 mV (20 mV step; inset) recorded from TRPC5-expressing cells. The currents were recorded in control solution ∼1 min after whole-cell formation, after 2 min of exposure to 100 μM tannic acid, and after 2 min of washing by control solution. Steady-state currents were measured at the end of the pulses as indicated by colored symbols atop the records. **B** The average currents at +200 mV obtained from protocol as in (A). Bar graph represents means +SEM (*n* = 6) **C** The average conductance-voltage plots normalized to the maximum response to +200 mV obtained under basal conditions. The lines connecting average data points (means ± SEM; *n* = 6) have no theoretical meaning. **D** Representative current traces in response to a 100-ms voltage step protocol from −80 to +200 mV (20 mV step) recorded from TRPC5-expressing cells. The currents were recorded in control solution ∼1 min after whole-cell formation, after 2 min of exposure to 100 μM genistein, and after 2 min of washing by control solution. Steady-state currents were measured at the end of the pulses as indicated by colored symbols atop the records. **E** The average currents at +200 mV obtained from protocol as in (D). Bar graph represents means +SEM (*n* = 10). **F** The average conductance-voltage plots normalized to the maximum response to +200 mV obtained in extracellular control solution (means ± SEM; *n* = 10). **G** Representative current traces in response to a 100-ms voltage step protocol from −80 to +200 mV (20 mV step) recorded from TRPC5-expressing cells. The currents were recorded in control solution ∼1 min after whole-cell formation, after 2 min of exposure to 100 μM genistin, and after 2 min of washing by control solution. Steady-state currents were measured at the end of the pulses as indicated by colored symbols atop the records. **H** The average currents at +200 mV obtained from protocol as in (G). Bar graph represents means +SEM (*n* = 5). **I** The average conductance-voltage plots normalized to the maximum response to +200 mV obtained in extracellular control solution (means ± SEM; *n* = 5). **J** Time course of representative currents of wild-type hTRPC5 co-expressed with Gα_i3_^Q204L^ subunit induced by 60-s-long exposure to the Cs-rich solution. 100 μM genistein was co-applied for 60 s. Right panel: Current-to-voltage relations from time points denoted by letters a-c in (J). **K** Time course of representative currents of WT hTRPC5 co-expressed with Gα_i3_^Q204L^ subunit induced by 60-s-long exposure to the Cs-rich solution. 100 μM genistin was co-applied for 60 s. Inset: Voltage ramp protocol used for activation. Right panel: Current-to-voltage relations from time points denoted by letters a-c in (K). **L** Maximal amplitudes of currents obtained in the presence of genistein (GEN) and genistin (GIN) at −100 mV and +100 mV, normalized to currents measured in control Cs-rich solution.

Another channel reported to play a role in sensory transduction is TRPC5, which underlies phospholipase C-, cold- and voltage-dependent currents in trigeminal neurons and odontoblasts (Bernal et al., 2021; Sadler et al., 2021). This channel is also present in gingival epithelial cells, where it participates in calcium-dependent keratinocyte maturation and barrier formation (Cai et al., 2005). We explored the effects of tannic acid (TA; 10 μM) on currents induced by depolarizing pulses from −80 mV to +200 mV measured from hTRPC5-expressing HEK293T cells in the absence of any agonist (Fig. 3A–C). In all cells examined, tannic acid applied for 2 min inhibited voltage-induced outward currents by about 40 % at +200 mV and this effect was partially reversible within the following 2 min. Thus, TRPC5 does not appear to be an excitatory channel that simply transduces information about the astringency of this substance from the periphery to the central nervous system. On the other hand, TRPC5, along with other targets, may mediate the well-known analgesic effect of tannic acid (Abbott et al., 2021).

### 3.6 Genistein potentiates TRPC5 activity induced by voltage and G-protein alpha subunit

It has been previously shown that genistein stimulates the activity of mouse TRPC5 and acts synergistically with La^3+^ (Wong et al., 2010). In our hands, a 2-min exposure to genistein (100 μM), but not genistin (100 μM), potentiated voltage-induced hTRPC5 conductance at +200 mV by 1.6-fold (Fig. 3D–I). To investigate the effect of genistein in the context of hTRPC5 activation downstream of G-protein coupled receptors (the primary physiological mechanism of TRPC5 activation), the constitutively active Gα_i3_^Q204L^ protein subunit was used. Co-transfected cells were exposed to the Cs-rich solution and voltage ramps of 500 ms duration from +100 mV to −100 mV were delivered periodically every 10 s from a holding potential of −60 mV in order to identify the intrinsic activity of TRPC5 with high permeability to Cs^+^ ions (Won et al., 2023). One-minute exposure to genistein (100 μM) induced a 3.2-fold increase in hTRPC5 outward current at +100 mV and an even 6.1-fold increase in inward current at −100 mV. In contrast, exposure to genistin caused no change in current responses (Fig. 3J–L). The original study by Wong et al. demonstrated that genistein acts on the TRPC5 channel relatively directly, and its action is unrelated to either tyrosine kinase inhibition or estrogen receptors (Wong et al., 2010). Our results support this hypothesis, because structurally closely related genistin, whose bulky sugar group hinders entry into tight hydrophobic binding pockets (Supplementary Fig. 5D), had no effect on the channel. We therefore further searched for a possible site of direct interaction between genistein and human TRPC5.

### 3.7 Predicted molecular binding modes of genistein at TRPC5

**Fig. 4:**
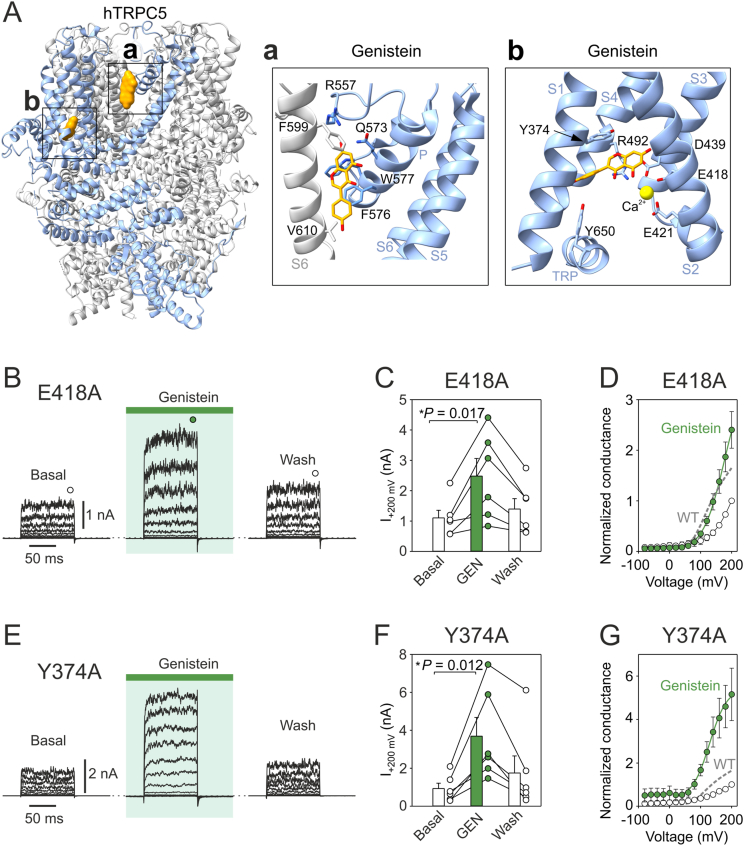
| Predicted binding sites for genistein in human TRPC5. **A** Using deep learning model Boltz-2, two binding sites for genistein were predicted. First in the lipid-/xanthine-binding site (panel a) and second in the inner cavity of the voltage sensor-like domain (VSLD, panel b). **B** Representative current traces in response to a 100-ms voltage step protocol from −80 to +200 mV (20 mV step) recorded from E418A-TRPC5-expressing cells. The currents were recorded in control solution ∼1 min after whole-cell formation, after 2 min of exposure to 100 μM genistein, and after 2 min of washing by control solution. Steady-state currents were measured at the end of the pulses as indicated by colored symbols atop the records. **C** The average currents at +200 mV obtained from protocol as in (B). Bar graph represents means +SEM (*n* = 6). **D** The average conductance-voltage plots normalized to the maximum response to +200 mV obtained under basal conditions. The data were fitted by Boltzmann equation over the interval +20 mV to +200 mV (solid lines; *n* = 6). Normalized average steady-state conductance obtained from wild-type TRPC5 expressing cells in the presence of genistein is shown as a gray dashed line for comparison. **E** Representative current traces in response to a 100-ms voltage step protocol from −80 to +200 mV (20 mV step) recorded from Y374A-TRPC5-expressing cells. The currents were recorded in control solution ∼1 min after whole-cell formation, after 2 min of exposure to 100 μM genistein, and after 2 min of washing with the control solution. Steady-state currents were measured at the end of the pulses. **F** The average currents at +200 mV obtained from protocol as in (E). Bar graph represents means +SEM (*n* = 6). **G** The average conductance-voltage plots normalized to the maximum response to +200 mV obtained in extracellular control solution. The data were fitted by Boltzmann equation over the interval +20 mV to +200 mV (solid lines; *n* = 6). Normalized average steady-state conductance obtained from wild-type TRPC5 expressing cells in the presence of genistein is shown as a gray dashed line for comparison.

Our initial predictions based on Boltz-2 suggested two potential binding sites for genistein in human TRPC5, one in the conserved lipid-, (−)-englerin A- and xanthine-binding site between the pore re-entrant helix and S5/S6 helices (Q573, F576, W577) (Chen et al., 2025; Porav et al., 2025; Song et al., 2021; Wright et al., 2020), and one inside the pocket of the voltage-sensor-like domain (VSLD), adjacent to the Ca^2+^ ion (Y374, D439, M442, R492), that overlaps with a previously identified riluzole- and clemizole-binding site (Song et al., 2021; Yang et al., 2022). Out of twenty predictions, the first position was identified in eleven cases, with the remaining predictions preferring the second position (Fig. 4A and Supplementary Fig. 5A). Both binding sites had similarly low predicted affinity values (affinity_pred_value of 1.43 ± 0.2 and 1.40 ± 0.2; *P* = 0.331) and binding probabilities (median 0.35 and 0.36; P = 0.254). The first identified site forms a highly conserved lateral hydrophobic fenestration which accommodates endogenously present diacylglycerol (DAG), a key physiological activator that controls the gating of most TRPC channel family members (Duan et al., 2019; Lichtenegger et al., 2018; Song et al., 2021; Storch et al., 2021; Wright et al., 2020). Therefore, next we included 4 molecules of DAG, 4 molecules of genistein and 4 calcium ions in the input file for Boltz-2. The ten predictions we ran were surprisingly consistent (Supplementary Fig. 5B and C). In line with previous structural studies (Duan et al., 2019; Chen et al., 2025; Porav et al., 2025; Song et al., 2021; Wright et al., 2020), all four DAG molecules bound to the lipid/xanthine-binding site. Calcium ions were coordinated invariably within the inner crevice of the sensor domain by negatively charged residues E418, E421, and D439. Genistein always bound in the sensor domain above the Ca^2+^ coordination site, contacting predominantly F414, D439, Y374, and H370. To further explore the possible role of this region, we ran additional unbiased 13 predictions with the wild-type and 15 predictions with the double mutant Y374A/E418A of the TRPC5 monomer and found a significant increase in the binding affinity of genistein (affinity_pred_value from 0.85 ± 0.03 to 0.78 ± 0.02; *P* = 0.030) (Supplementary Fig. 5E and F). Using whole-cell electrophysiology, we experimentally tested the genistein sensitivity of two hTRPC5 constructs, E418A and Y374A (Fig. 4B–F). The currents induced by depolarizing pulses from −80 mV to +200 mV measured in the absence of any agonist were greatly suppressed in E418A and abolished to the level of non-transfected HEK cells in Y374A, as we reported previously (Zimova et al., 2022). However, genistein applied for 2 min at a concentration of 100 μM dramatically potentiated voltage-induced outward currents by about 2.5- in E418A and 5-fold in Y374A at +200 mV and this effect was partially reversible within the following 2 min. The application of genistein caused Y374A to change from essentially insensitive to a voltage-sensitive channel characterized by the half-maximal voltage (*V*_50_) of steady-state activation of 128.5 ± 4.6 mV and the apparent number of gating charges (*z*) of 0.90 ± 0.05 *e*_0_ (*n* = 6). These values were not significantly different from those observed in the wild-type channel (129.0 ± 2.3 mV; *P* = 0.921 and 0.83 ± 0.07 *e*_0_; *P* = 0.449; *n* = 10). Together, these results indicate that genistein helps to stabilize the open state of hTRPC5 during voltage-dependent gating and suggest that the inner cavity of the voltage-sensor-like domain may represent a potential binding site for this compound.

### 3.8 MUC1 may directly interact with TRPC5 and TRPV2 and modulate their currents

A recent hypothesis on the molecular origin of astringency suggests a key role for transmembrane mucin MUC1, a heavily glycosylated single transmembrane protein, abundantly expressed on the surface of oral epithelial cells (Canon et al., 2021). Together with other salivary mucins, it participates in the formation of a protective lubricating layer on the oral mucosa. When this layer is disrupted by, e.g., an astringent compound, the intracellularly oriented C-terminal subunit of MUC1 activates and passes this information further via interaction with specific interacting partners (Kufe, 2020). From the TRP ion channel family, so far only Ca^2+^ selective TRPV5/6 have been shown to be functionally linked to MUC1 (Al-Bataineh et al., 2023). MUC1 enhances TRPV5/6 cell surface expression by reducing endocytosis. We asked whether MUC1 expression has an impact on human TRPC5 and TRPV2 ion channel currents.

**Fig. 5:**
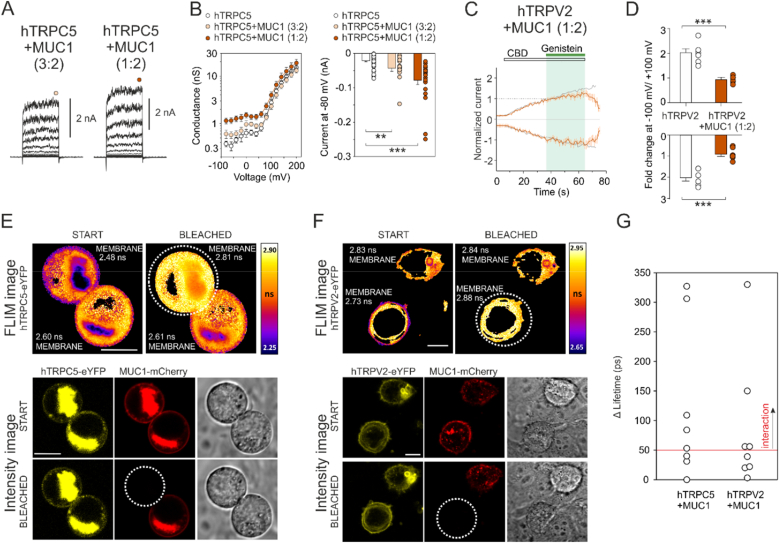
| Transmembrane mucin MUC1 functionally affects and likely interacts with hTRPC5 and hTRPV2. **A** Representative current traces in response to a 100-ms voltage step protocol from −80 to +200 mV (20 mV step; inset) recorded from TRPC5 and MUC1-expressing cells in different ratios. **B** Left: Average conductance of WT hTRPC5 (*n* = 43) and WT hTRPC5 with MUC1 expressed in the ratios 3:2 (*n* = 14) and 1:2 (*n* = 23) obtained from recordings as in (A). Right: Average currents at −80 mV obtained from protocol as in (A). **C** Average normalized whole-cell currents recorded from a HEK293T cell expressing hTRPV2 with MUC1 in the ratio 1:2, measured at −100 mV and +100 mV. Currents were normalized to maximum amplitudes elicited after 30s application of CBD. Overlaid average normalized currents from HEK293T cells expressing only hTRPV2, induced by CBD alone (gray lines). **D** Relative change in peak currents after 30s application of genistein versus current extrapolated by fitting the CBD currents with a single-exponential function. (*n* = 6 and 7). ∗∗*P* < 0.01, ∗∗∗*P* < 0.001; Student's two-tailed *t-*test. Error bars represent standard error of the mean (SEM). **E, F** Fluorescence lifetime images (FLIM) and intensity images of cells expressing hTRPC5 (E) or hTRPV2 (F) with MUC1 in HEK293T cells. FLIM images are taken before (left, START) and after (right, BLEACHED) photodestruction of mCherry fluorescence acceptor. The photodestructed area is marked by the dashed line, bar is 10 μm. **G** Graphic representation of repeats obtained with several independent cell samples. Interaction is considered positive it the Δ−Lifetime is ≥ 50 ps.

The addition of an expression plasmid encoding human MUC1 to the transfection mixture at a 1:2 ratio (TRPC5:MUC1) significantly increased basal inward currents at −80 mV compared to the expression of hTRPC5 alone (median; from −17 pA to −57 pA; *P* < 0.001; *n* = 43 and 14; Fig. 5A and B). The addition of less MUC1 (3:2 - TRPC5:MUC1) did not cause such an increase, although a significant rise in inward current at −80 mV was still detected (median −40 pA; *P* = 0.009; *n* = 14). The amplitudes of voltage-activated currents at +200 mV did not significantly differ from the control in the presence of MUC1 (*P* = 0.936 and *P* = 0.510 for TRPC5:MUC1 of 1:2 and 3:2, respectively). hTRPV2 currents were also affected by the co-transfection of MUC1. In contrast to cells expressing only hTRPV2, genistein did not significantly increase hTRPV2-mediated CBD-induced currents at both negative and positive membrane potentials when MUC1 was added to the transfection mixture at a ratio of 1:2 (Fig. 5C and D). After 30 s of genistein exposure, CBD-induced responses changed 0.9 ± 0.1-fold at −100 mV and 1.0 ± 0.1-fold at +100 mV (*n* = 7), which corresponds to the extrapolated increase when CBD was applied alone (compare Fig. 1D–J). The loss of the potentiating effect of genistein may be a result of direct interaction of MUC1, which apparently can compromise the flexibility of the transmembrane region of the channel (Supplementary Fig. 6). We also tested whether the presence of MUC1 affects heat-induced hTRPV2 currents (Supplementary Fig. 3D–I). CBD induced large currents in co-transfected cells, which were inhibited by heat in the range of 30–50 °C (Supplementary Figs. 3D, G, H). Although this is an interesting finding that has not yet been described in human TRPV2 itself, further studies are needed to draw definitive conclusions about the effects of MUC1. Nevertheless, taking together, our observations suggest that the presence of MUC1 affects the function of both hTRPC5 and hTRPV2 in HEK293T cells.

To investigate whether MUC1 is capable of direct interaction with hTRPC5 or hTRPV2, we performed combined fluorescence imaging and Förster's resonance energy transfer (FLIM-FRET) microscopy experiments, which evaluate the distance between the donor and acceptor in the order of nanometers. HEK293T cells were co-transfected with hTRPC5-eYFP or hTRPV2-eYFP as a fluorescence donor and MUC1-mCherry as a fluorescence acceptor. When the donor and acceptor are in close proximity, a fraction of the energy of the donor's excited state is transferred to the acceptor in a radiation-free manner, which leads to a decrease in the lifetime of the donor's fluorescence. First, the initial FLIM image was taken. Then, the mCherry acceptor in a selected cell was photodestructed by strong 561 nm light irradiation. The FLIM image after photobleaching was taken, and the donor fluorescence lifetime after acceptor photobleaching was compared with the initial one. In case of interaction, the acceptor photobleaching leads to an increase in donor fluorescence lifetime (Herman and Lakowicz, 2014).

Five of the eight measured cells that co-expressed hTRPC5-eYFP with MUC1-mCherry showed a sufficient increase (≥50 ps) in eYFP fluorescence lifetime after mCherry photobleaching, confirming the TRPC5-MUC1 interaction at the plasma membrane. In the case of hTRPV2-eYFP with MUC1-mCherry, an interaction could be observed in four of the eight measured cells (Fig. 5E–G). These results suggest that both TRPC5 and TRPV2 are able to maintain spatial proximity with MUC1, likely through direct interactions (Supplementary Fig. 6).

## 4 Discussion

Our study identifies TRPV2 and TRPC5 ion channels as previously unknown molecular sensors for three structurally distinct representatives of astringent compounds, including auto-oxidation products of (−)-epigallocatechin-3-gallate, tannic acid and genistein. Astringency, as a tactile sensation, involves various sub-qualities mediated by different mechanisms at the molecular level, occurring in the oral epithelium and mucous membranes of the tongue, cheeks, gums, and palate, as well as the free nerve endings of the mechanically and chemically sensitive trigeminal nerves (Canon et al., 2021; Wang et al., 2024). Although the tip of the tongue is one of the most sensitive areas of the body to mechanical stimuli - more so than the fingertips (Miles et al., 2018; Trulsson and Essick, 1997) - the neural basis for its high tactile acuity remains largely unknown (Liu et al., 2022;Simon and Gutierrez, 2017; Zhang et al., 2024). Current research suggests that the main anatomical structures responsible for the high lingual sensitivity to tactile stimuli are fungiform papillae, the taste-bud-holding structures, convergently innervated by multiple trigeminal afferents expressing mechanosensitive Piezo-2 ion channels (Zhang et al., 2024). Our experiments tested the general possibility that the transmembrane mucin MUC1 affects TRPC5 and TRPV2 channel function in oral mucosa/epithelial taste cells. In this context, it is important to note that the presence of TRPC1/3/4/5 channel transcripts was recently demonstrated in taste cells isolated from the circumvallate papillae (Cherkashin et al., 2023); however, the role of TRPC5 awaits further investigation. The expression of MUC1 improves the formation of the mucosal pellicle and results in the presence of more hydrophobic and more charged areas at the surface of cells (Aybeke et al., 2019), which may be the physicochemical mechanism through which MUC1 affects transmembrane proteins such as TRPV5 and TRPV6 (Al-Bataineh et al., 2023). Mechanical gating also likely represents a unifying activation mechanism of polymodal TRP channels (Liu and Montell, 2015), which are found in somatosensory neurons from the trigeminal ganglia and are thought to integrate mechanical, chemesthetic and temperature stimuli. Phenolic phytochemicals are particularly promiscuous modifiers of membrane protein function, and their effects on these channels may partly be mediated by the membrane bilayer (Ingólfsson et al., 2014). In this context, it is therefore logical to search for temperature- and mechanically sensitive sensory ion channels whose tissue distribution and activity prime them for a role in taste detection or modulation.

Several other TRP channels have been studied in relation to their possible role in detecting astringency. A recent study by Kim and Simons (2024) on human subjects found that TRPV1 and TRPA1 are unlikely to play a pivotal role, as experiments relying on desensitization of these channels using their specific ligands suggest (Kim and Simons, 2024). This complex and thorough research may need further studies because desensitization induced by one type of agonist might not eliminate sensitivity to another (Novakova-Tousova et al., 2007). Another study demonstrated that tannic acid triggers an influx of Ca^2+^ without releasing Ca^2+^ from internal stores in endothelial cells (Tsai et al., 2020). This Ca^2+^ influx can be suppressed by non-specific Ca^2+^ and TRP channel blockers, as well as by the TRPC4/C5 and TRPM4 channel inhibitors (M084 and 4-Chloro-2-[[2-(2-chlorophenoxy)acetyl]amino]benzoic acid). Our results show that, instead, human TRPC5 is inhibited by tannic acid and probably does not have a major role in transducing its astringency. Additionally, as TRPM4 excludes divalent cations, making it distinct from many other TRP channels (Nilius et al., 2003), the identity of a channel mediating these responses thus remain undisclosed. Tannic acid is also a potent activator of voltage-gated potassium K_V_7.2/7.3 channels that contribute to the M-current in sensory neurons (Abbott et al., 2021). Activating these channels lowers neuron excitability, making it also unlikely that this effect is responsible for transducing the astringent signal.

In this study, we show for the first time that human TRPV2 can be activated by heat, meaning that under certain conditions, temperature can modulate the responses of this channel. The effects of temperature on astringency is known to depend on the type of astringent compounds (Bajec et al., 2012). *In vitro* studies propose that in the case of EGCG, the intensity of astringency results from interactions with the salivary proteins mucin and α-amylase, which are temperature-dependent (Ye et al., 2021). On the other hand, a study in human subjects showed that lingual sensitivity to roughness, which is a perception associated with the ability to discriminate astringency (Linne and Simons, 2017), decreased at low temperatures. The cooling substance Evercool 190 that activates the cold-sensitive TRPM8 channels, however, did not alter it (Ricci et al., 2024). The intensity of perception of some astringents may be affected by temperature due to both physiological and physicochemical mechanisms (Bajec and Pickering, 2008). The sensitivity of human orthologues of TRPV2 to heat (demonstrated in this study) and TRPC5 to cold (Ptakova et al., 2022; Zimmermann et al., 2011) may contribute in different ways to the effect of various astringent substances - our study could inspire further experiments in humans in this context.

Our data suggest that temperature sensitive epithelial and neuronal ion channels TRPV2 and TRPC5 may play a role in transducing some astringent stimuli through direct chemical interactions. This aligns with previous reports implicating TRPV1 and TRPA1 in detecting astringency (Kurogi et al., 2012, 2015), and our findings also show that responses vary depending on the species. This makes detailed investigations, which ultimately must involve human subjects, even more complex (Thibodeau et al., 2020). On the other hand, broader questions emerge about how diet - shaped by the surrounding environment - affects evolutionary pressures across different animal species. The opposing effects of oxi-EGCG and tannic acid on human and rat TRPV2 reinforce the view that these compounds engage multiple channel regions. The differences observed likely do not stem from a highly conserved interaction site but instead from non-conserved, allosterically coupled domains that ultimately influence channel gating (Fricke et al., 2025; Juvin et al., 2007; Neeper et al., 2007).

There are limitations to our study. While Boltz-2 provides useful predictions of ligand-channel interactions, experimental structural validation (e.g., cryo-EM with bound ligand) will be essential to confirm binding poses and allosteric pathways. Our electrophysiological recordings were performed in heterologous HEK293T cells, which lack the full complement of native signaling pathways, and they do not fully recapitulate *in vivo* gating behavior. Future studies using native sensory tissues or human-derived organoids could further elucidate the physiological relevance of our findings. The striking species specificity of the effects of astringents on TRPV2 observed in this study is definitely a challenge that needs to be tackled first.

The role of TRPV2 and TRPC5 in mediating the effects of the phytochemicals described here may have important implications that extent beyond astringency. From a physiological perspective, both channels are expressed in non-neuronal cells such as epithelial cells, immune cells, and cancer cells, where their activation could influence barrier function, inflammation, or cell survival (He and Ma, 2016; Hironaka et al., 2014; Chinigò et al., 2020; Khare et al., 2024; Siveen et al., 2020). There is extensive literature on the health-promoting effects associated with phenolic phytochemicals (see e.g. (Wei et al., 2023) and references therein). Polyphenols like tannic acid, EGCG and genistein are known for their antioxidant, anticancer, and antimicrobial properties, and their ability to modulate TRP channels may contribute to these effects as well. For example, genistein is widely recognized for its estrogenic, antioxidant, and anti-inflammatory properties, including its pleiotropic anticancer effects (Dixon and Ferreira, 2002; Naponelli et al., 2025). The potentiation of human TRPV2 and TRPC5 by genistein and modulation of these channels in mucin 1-rich environments could influence epithelial permeability or nociception during exposure to dietary astringents or during inflammation. On the other hand, local administration of EGCG effectively suppresses the excitability of nociceptive primary and secondary neurons in rats (Uchino et al., 2023; Utugi et al., 2025a, 2025b), suggesting its potential as a local anesthetic with minimal side effects. It is important to acknowledge that results from animal models may not always directly translate to humans. The polymodal nature of activation of TRPV2 and TRPC5 could help clarify different theories of astringent perception, including tactile and chemosensory pathways, providing a more nuanced understanding of how we perceive this complex sensation.

## 5 Conclusions

In conclusion, we identify TRPV2 and TRPC5 as previously unrecognized targets of astringent polyphenolic compounds. These channels are modulated through distinct binding pockets and modulatory mechanisms, including temperature gating, G-protein pathways, and structural interaction with mucin 1. Our findings not only provide new insight into the molecular basis of astringency detection but also expand the pharmacological landscape of TRP channels, suggesting potential roles in sensory modulation, epithelial physiology, and natural product-based therapeutics.

## CRediT authorship contribution statement

Anna Kadkova: Data curation, Formal analysis, Investigation, Writing – original draft. Kamila Kosinova: Data curation, Investigation, Formal analysis. Marketa Klouckova: Data curation. Ivan Barvik: Investigation, Methodology, Software, Validation. Dita Strachotova: Data curation, Investigation, Methodology, Formal analysis. Lucie Zimova: Investigation, Formal analysis, Visualization, Supervision, Writing – original draft. Viktorie Vlachova: Conceptualization, Data curation, Formal analysis, Supervision, Funding acquisition, Validation, Visualization, Methodology, Writing – original draft, Writing – review and editing.

## Funding

This research was supported by the 10.13039/501100001824Czech Science Foundation (24-10147S) and the Ministry of Education, Youth and Sports of the Czech Republic, project INTER-EXCELLENCE II (LUC24020; COST Action CA22161 FLAVOURsome).

## Declaration of competing interest

The authors declare that they have no known competing financial interests or personal relationships that could have appeared to influence the work reported in this paper.

## Data Availability

All data generated or analysed during this study and its supplementary information files are available from the corresponding author on reasonable request.
