# Supplementary material for: TRPV2 and TRPC5 are potential targets for astringent phytochemicals
Source: Curr Res Food Sci. 2026 Jan 10;12:101306. doi: 10.1016/j.crfs.2026.101306 (PMC12830184; doi:10.1016/j.crfs.2026.101306)
Supplement: Multimedia component 1 [file mmc1.docx]

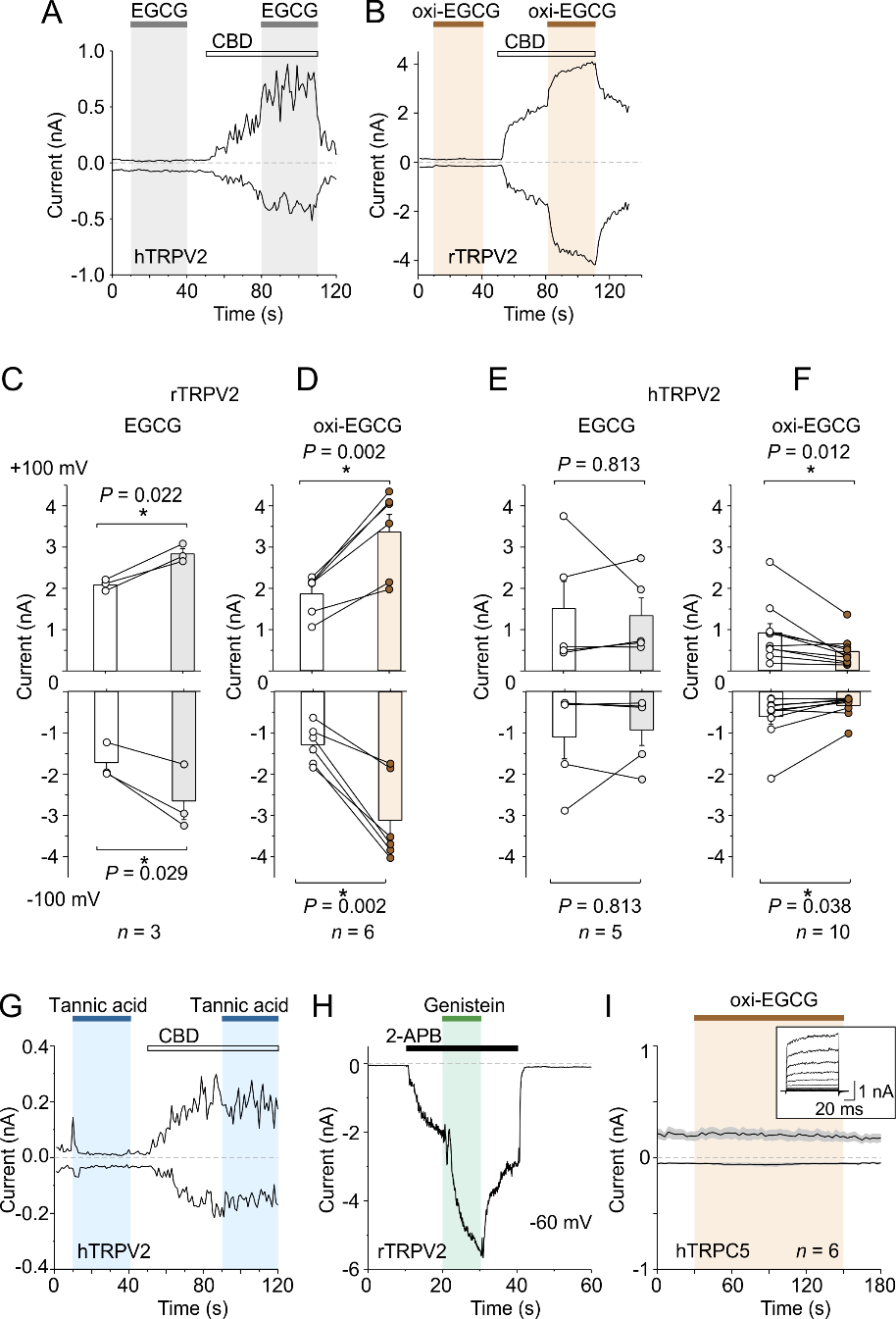


**Supplementary Figure 1.** Astringent compounds modulate activity of rat TRPV2 (rTRPV2) and human TRPV2 (hTRPV2). **A**, **B** Time course of representative whole-cell currents recorded from HEK293T cells expressing human or rat TRPV2, measured at -100 mV and +100 mV. EGCG (100 µM) or the auto oxidation products of EGCG (oxi-EGCG; 100 µM; incubated for longer than 70 min) were applied alone and then in the presence of cannabidiol (CBD; 30 µM). CBD was pre-applied prior to its co-application with an astringent. A ramp pulse was periodically applied every 1 second for 500 ms. **C**-**F** Statistics of the effects of EGCG and oxi-EGCG in rTRPV2 and hTRPV2. Current amplitudes were measured at -100 mV and at -100 mV in the presence of CBD+EGCG or oxi-EGCG after 30 s and compared with the amplitude of currents induced by CBD alone. **G** Currents induced by tannic acid (10 µM) in hTRPV2. **H** Currents induced by 2-APB (300 µM), mediated by rTRPV2, measured at -60 mV were potentiated by genistein (100 µM). **I** Average currents measured at -100 mV and +100 mV in the absence and presence of 100 µM oxi-EGCG in HEK293T cells expressing hTRPC5. The insert shows average voltage-dependent currents induced by depolarizing pulses from -80 mV to +200 mV recorded from 6 cells, which were measured to demonstrate strong functional expression of the channels.


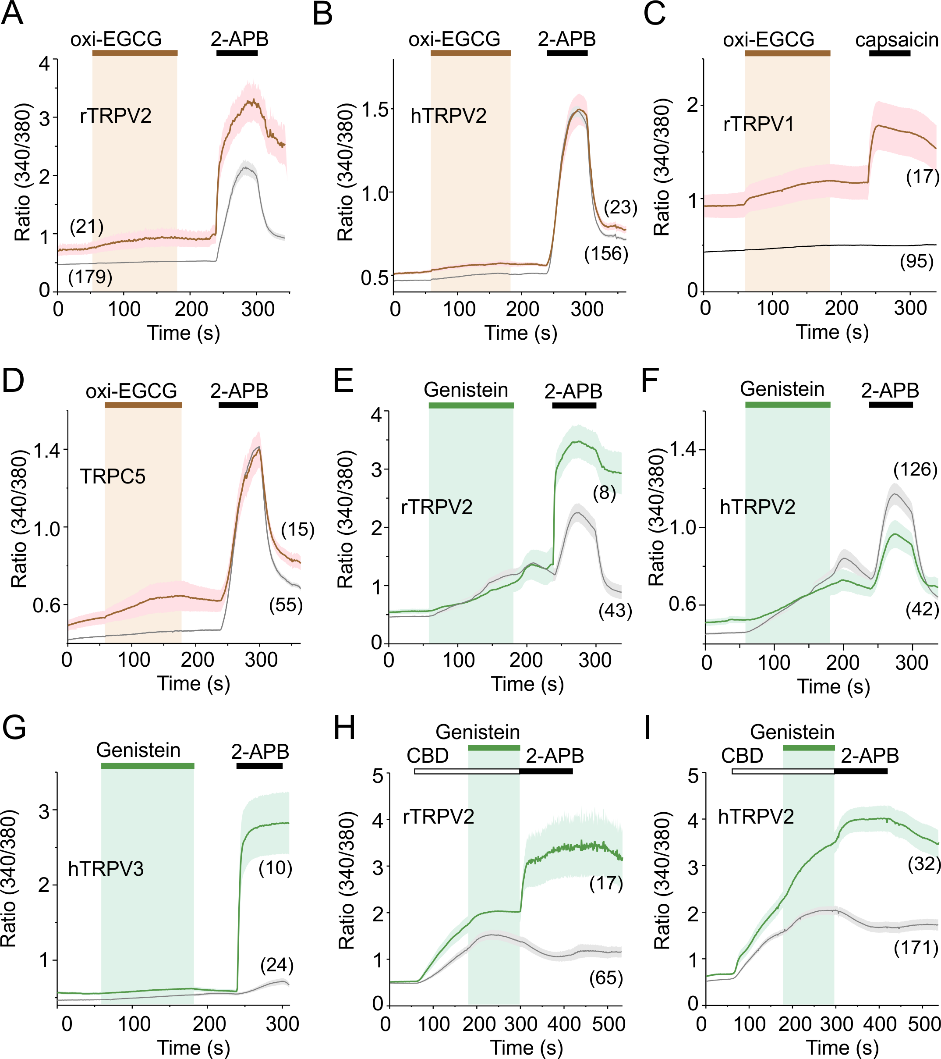


**Supplementary Figure 2.** Astringent compounds modulate activity of rat TRPV2 (rTRPV2), human TRPV2 (hTRPV2) and human TRPC5. **A**-**I** Ca^2+^ imaging experiments in HEK293T cells transfected with indicated constructs; mCherry was used as a marker of positively transfected cells. Responses from cells not expressing the fluorescent marker are denoted by gray lines and light-gray envelopes. The auto oxidation products of EGCG (oxi-EGCG; 100 µM; incubated for longer than 70 min). The concentration of 2-APB was 1 mM in A-G, and 100 µM in H and I. CBD and genistein were used at concentrations 30 µM and 100 µM. The darker curves are the mean and lighter-colored envelopes the standard error (*n* indicated in parentheses). Note in panel F that 2-APB responses are significantly lower in cells expressing hTRPV2. We currently have no explanation for this observation.


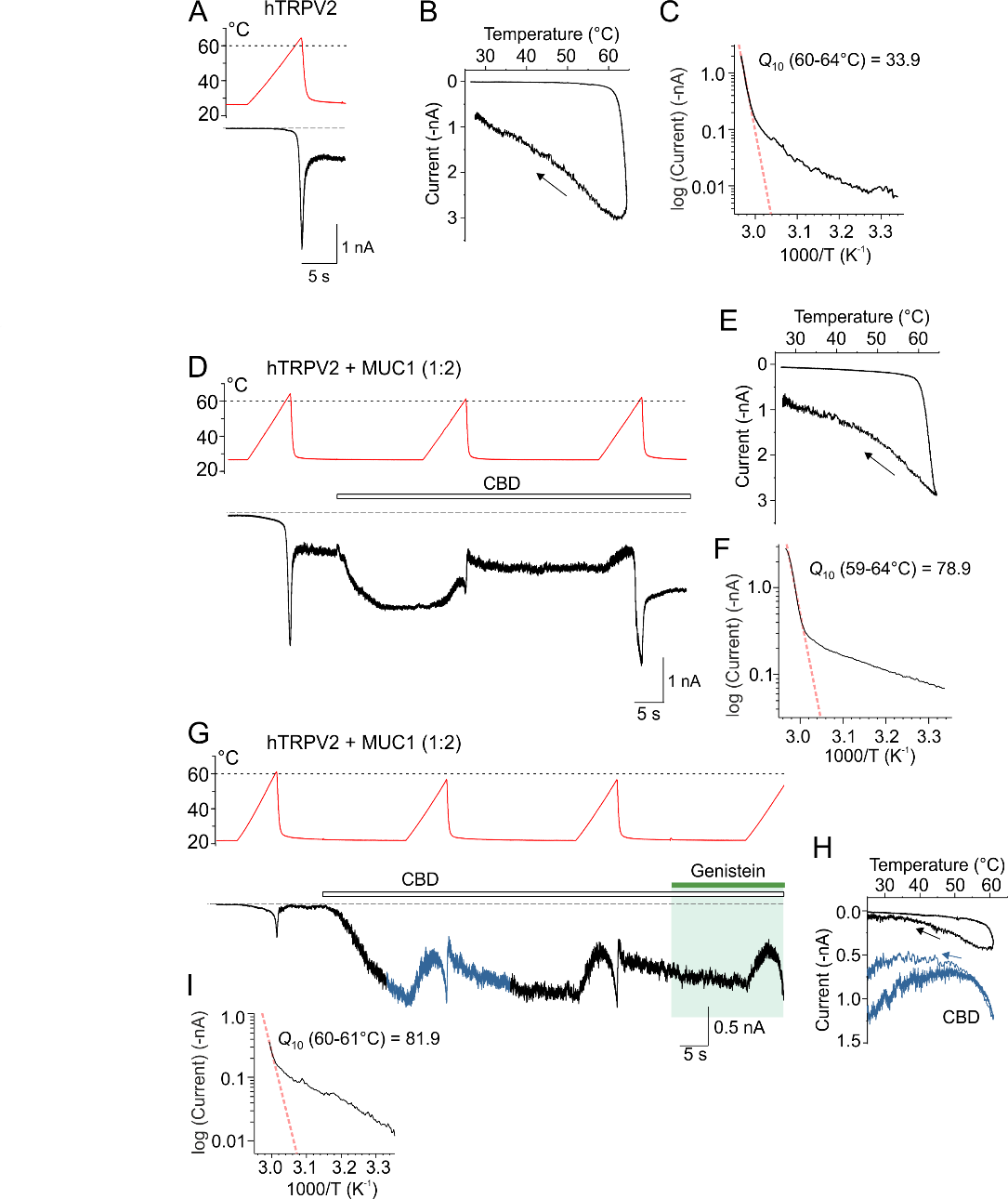


**Supplementary Figure 3.** Human TRPV2 can be activated by heat exceeding ~56-60°C. **A** Inward current mediated by hTRPV2, measured at a holding potential of -60 mV. **B** Current-temperature relationship for the response shown in A. **C** Arrhenius plot for the current shown in A. Red dotted line indicates the temperature range used for the *Q*_10_ estimation. **D** Whole-cell current measured from a representative HEK293T cell co-expressing hTRPV2 and MUC1 at -60 mV. Heat apparently inhibited inward current induced by cannabidiol (CBD; 30 µM). In the presence of control Ca^2+^-free bath solution, the cells frequently did not tolerate the repeated heat ramps for more than a few minutes during which they visibly deteriorated (see the large inward current at the end of the recording). **E and F** Current-temperature relationship and Arrhenius plot for the first heat response shown in D, obtained in control Ca^2+^-free bath solution. **G** Whole-cell current measured from a representative HEK293T cell co-expressing hTRPV2 and MUC1 at -60 mV. **H** Current-temperature relationships for the ascending and descending phases of heat response in control bath solution (black line) and in the presence of CBD (blue line). Note that after cooling, the current response obtained in control solution returns to almost its original level. This is probably because the temperature only slightly exceeded 60°C. **I** Arrhenius plot for the first heat response shown in G.


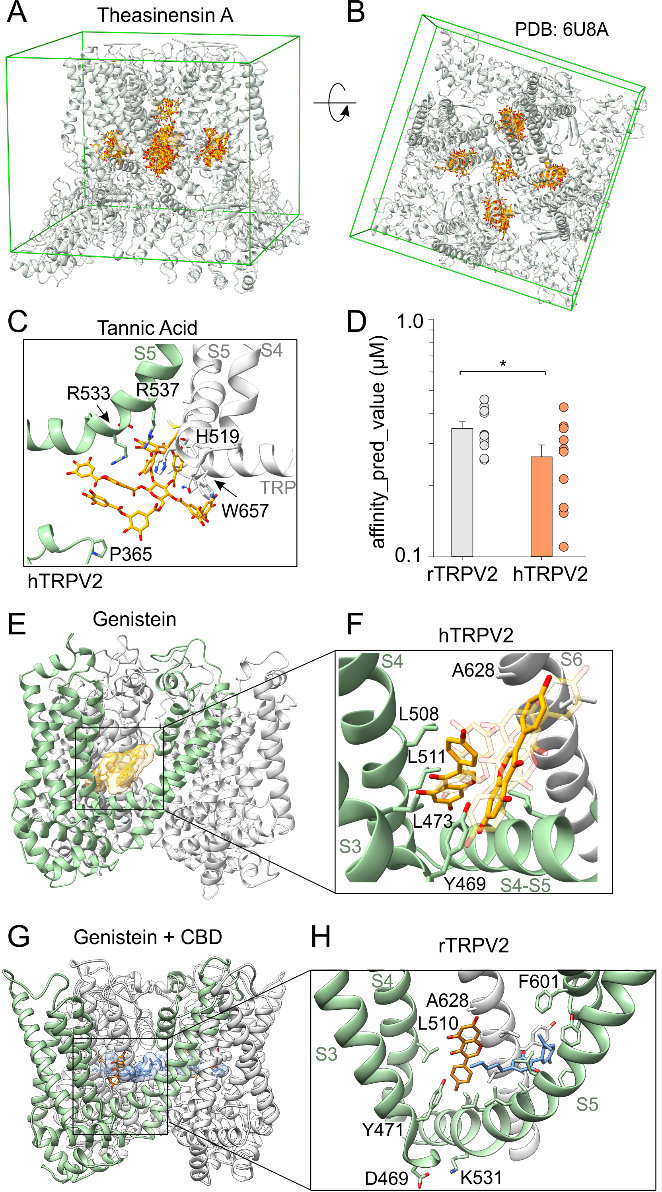


**Supplementary Figure 4**. Molecular interactions of TRPV2 with theasinensin A, tannic acid and genistein. **A, B** Interaction site for theasinensin A predicted by Autodock Vina using the CBD-bound full-length structure of rat TRPV2, state 1 (PDB ID: 6U8A). 78 out of the 80 output poses exhibited conformations located within the pocket formed by the positively charged residues H521, R535, and R539, located at the intracellular interface between S5 and the S4–S5 linker of two monomers. Two remaining output positions were located inside the channel pore. **C** Tannic acid (TA) interaction site at hTRPV2, predicted by Boltz-2. **D** The average predicted binding affinity for TA in rat and human TRPV2 orthologues derived from 10 independent predictions by Boltz-2. **E, F** Variability of genistein position within the vanilloid pocket of human TRPV2 predicted by Boltz-2. **G, H** Exemplary Boltz-2 prediction of genistein position at rTRPV2 including four molecules of cannabidiol (blue sticks) and four molecules of genistein (orange sticks).


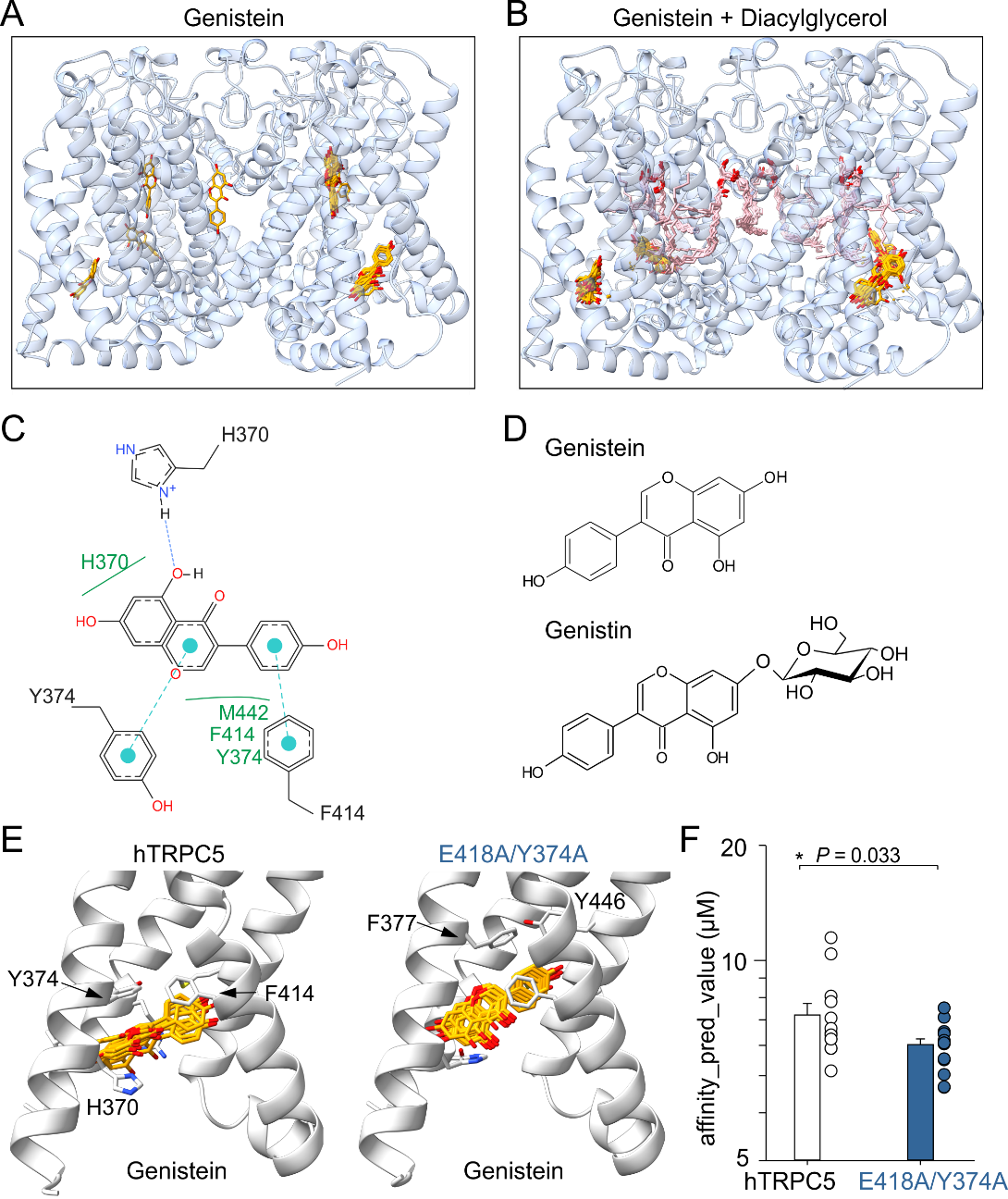


**Supplementary Figure 5.** Molecular interactions of hTRPC5 with genistein predicted by Boltz-2. **A** Interactions predicted from 20 simulations. Out of twenty predictions, the position in the lipid/xanthin domain was identified in eleven cases, with the remaining simulations preferring the position within the inner cavity of the voltage-sensor-like domain (VSLD). **B** The output from ten independent Boltz-2 predictions, that included 4 molecules of diacylglycerol, 4 molecules of genistein and 4 calcium ions. **C** Molecular interactions of genistein, 2D view (created by PoseView). **D** Comparison of the chemical structures of genistein and genistin. **E** Conformations of genistein in VSLD of wild-type hTRPC5 and the E418A/Y374A mutant predicted by Boltz-2. **F** Statistics of the predicted affinity values from 10 independent runs for each construct.

**
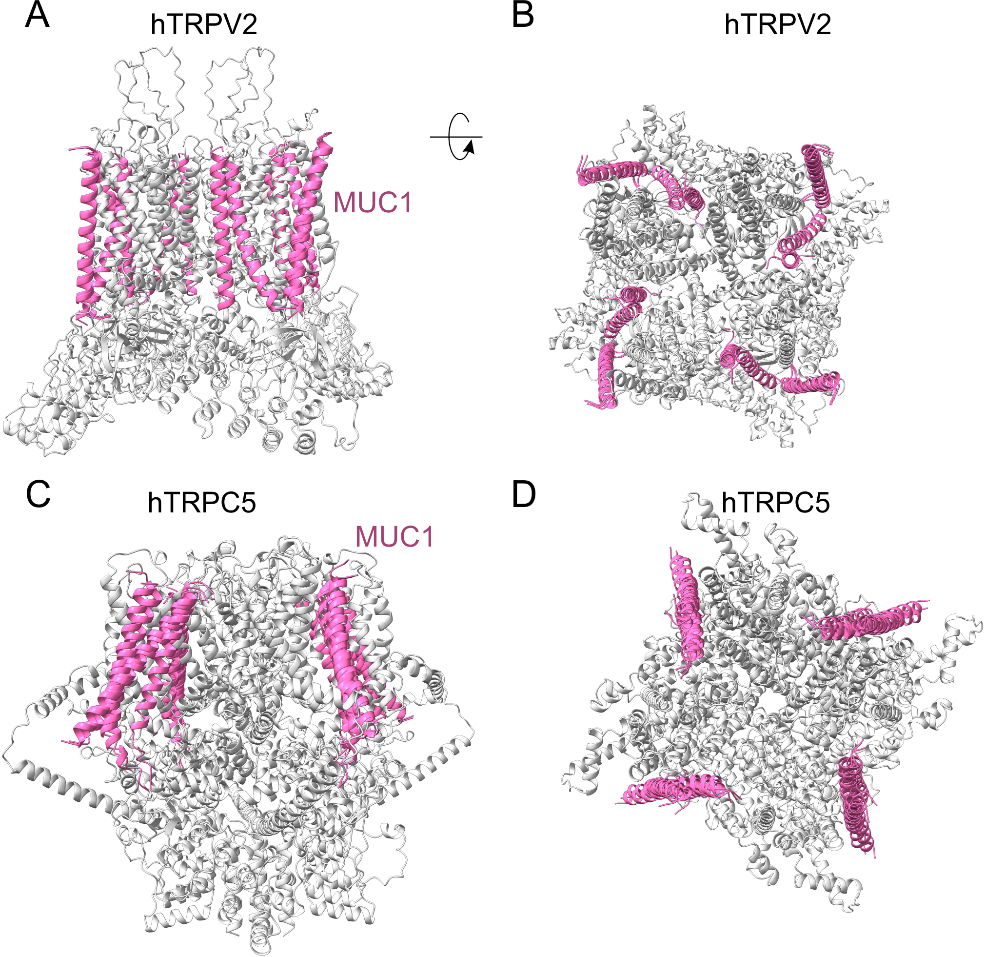
**

**Supplementary Figure 6.** AlphaFold predictions of hTRPV2 (**A**, **B**) and hTRPC5 (**C**, **D**) interaction with the transmembrane part of human MUC1 („VPGWGIALLVLVCVLVALAIVYLIALAVCQCRRKNYGQL“). Extensive interactions in the transmembrane region and in membrane-proximal regions suggest a significant influence of MUC1 on the function of both receptors.
